# Evaluation of the specificity of [^18^F]fludarabine PET/CT in a xenograft model of follicular lymphoma: comparison with [^18^F]FDG and impact of rituximab therapy

**DOI:** 10.1186/s13550-015-0101-7

**Published:** 2015-04-14

**Authors:** Narinée Hovhannisyan, Stéphane Guillouet, Fabien Fillesoye, Martine Dhilly, Delphine Patin, Françoise Galateau, Michel Leporrier, Louisa Barré

**Affiliations:** CEA, DSV/I2BM, LDM-TEP group, GIP Cyceron, Bd Henri Becquerel, BP 5229, 14074 Caen, Cedex France; Université de Caen Basse-Normandie, Esplanade de la Paix, 14032 Caen, France; CNRS, UMR ISTCT 6301, LDM-TEP group, GIP Cyceron, Bd Henri Becquerel, BP 5229, 14074 Caen, Cedex France; CHU de Caen, Service d’Anatomie Pathologique, Av. de la Côte de Nacre, BP 95182, 14033 Caen, Cedex France

**Keywords:** [^18^F]Fludarabine, PET/CT, Imaging, Lymphoma, Rituximab

## Abstract

**Background:**

[^18^F]Fludarabine is a novel positron emission tomography (PET) radiotracer for imaging lymphoma. The purpose of this preclinical study was to evaluate the robustness of [^18^F]fludarabine during rituximab therapy. In addition, a comparison was made between [^18^F]fludarabine and [^18^F]fluorodeoxyglucose ([^18^F]FDG) with regard to their concordance with histologically derived data.

**Methods:**

CB17-SCID mice bearing human follicular DOHH-2 lymphoma were treated once weekly with rituximab (10 mg/kg) or physiological saline over 3 weeks. To obtain the tracer uptake in the metabolically active volume of the tumour (MAVT), a background-level threshold was applied to the volume of interest (VOI) defined on computed tomography (CT) image. The tumour uptake analysis was performed with MAVT-based segmentation for data analysis of sequential [^18^F]fludarabine PET/CT studies and with total tumour-based segmentation for comparison with histologically derived data.

**Results:**

The correlation between the MAVT and [^18^F]fludarabine accumulation (%ID) in those viable tissues was equally significant for both vehicle- or rituximab-treated mice; for these latter, the presence of lymphoid tissues at the end of imaging sessions was confirmed histologically. A stronger correlation was demonstrated between quantitative values extracted from [^18^F]fludarabine-PET and histology (*r*^2^ = 0.91, *p* < 0.001) when compared to [^18^F]FDG-PET (*r*^2^ = 0.55, *p* = 0.03).

**Conclusions:**

[^18^F]Fludarabine uptake in the follicular lymphoma model compared favourably with [^18^F]FDG in terms of specificity for PET imaging and also remained robust for persistent viable tissues following rituximab therapy. [^18^F]Fludarabine PET/CT may be a promising approach to evaluate lymphoma, including their surveillance during therapy.

**Electronic supplementary material:**

The online version of this article (doi:10.1186/s13550-015-0101-7) contains supplementary material, which is available to authorized users.

## Background

The non-Hodgkin lymphomas (NHLs) are a greatly diverse family of disorders characterized by malignant proliferation of B or T lymphocytes [1,2]. PET has been proven to be the only imaging technique able to encompass all the information yielded by conventional morphological techniques (lymphangiography, computed tomography, ultrasonography, magnetic resonance imaging). PET furthermore provides essential information for the assessment of chemosensitivity and the planning of radiotherapy. This tomographic technique computes the three-dimensional distribution of radioactivity based on the annihilation of the photons that are produced in tissue by positron-emitting labelled radiotracers. PET, based on the [^18^F]FDG method, is of proven usefulness in the diagnosis and monitoring of therapy in patients with lymphoma. However, the specificity of [^18^F]FDG uptake has been questioned because of its behaviour similar to glucose metabolism, which may increase indiscriminately in benign conditions such as inflammatory or infectious processes [3-5]. Increasingly, research is oriented towards radiotherapy planning and it will be important that areas, such as the edge detection of tumours, can be readily integrated into the parameters for radiotherapy. To achieve this goal, more specific tracers, which nonetheless maintain all of the positive characteristics of [^18^F]FDG, are needed.

[^18^F]Fludarabine is a novel PET radiotracer which was developed for lymphoma imaging. The radiosynthesis of this tracer, as well as its biodistribution and dosimetric studies in animal models, has been reported recently [6,7]. In the present study, we evaluated the reliability of [^18^F]fludarabine during rituximab therapy in a follicular lymphoma model (indolent B-cell NHL) in xenografted mice. The question posed was whether or not the specificity of [^18^F]fludarabine for lymphoid tissues is modified by the treatment. Furthermore, we compared [^18^F]fludarabine with [^18^F]FDG which, in spite of the limitations of the latter, is currently the tracer of choice for imaging lymphoma.

## Methods

### Radiochemical synthesis

[^18^F]Fludarabine was synthesized on a TRACERlab™ FX F-N module as described previously [6]. Mean injected radioactivity was approximately 11 MBq, and molecular quantity of injected fludarabine was approximately 0.038 nmol. [^18^F]FDG was purchased from the commercially available source (Cyclopharma S.A., Caen, France).

### Animal model

The animal investigations were performed under the European directive (86/609/EU) as enacted in national legislation. The licence to investigate was given to M. Dhilly (approval 14-54) in authorized housing (the Biological Resources Centre (CURB) of the University of Caen (approval A14-118-015)) immediately adjacent to the laboratories of experimentation and imaging (GIP Cyceron; approval D14-118-001). Permission was sought and obtained for all experimental procedures from the regional committee on animal ethics (approval CENOMEXA 1112-26). A total of 18 mice were employed in the investigation.

CB17-SCID female mice bearing human DOHH-2 lymphoma cells (EBV negative) were purchased from Oncodesign (Dijon, France). DOHH-2 cells were established from the pleural effusion of a 60-year-old man with refractory immunoblastic B cell lymphoma progressed from a follicular centroblastic/centrocytic lymphoma. One million DOHH-2 cells, from exponentially growing *in vitro* cultures, were subcutaneously injected into the right flank.

The mice were kept in specific pathogen-free housing, in a 12/12-h day/night cycle, at 22°C and had access to sterilized laboratory chow and water *ad libitum*. At the time of the experiments, the mice were approximately 9 weeks old. The mean tumour volume at the initiation of therapy was approximately 150 mm^3^ (calliper).

For sequential studies with [^18^F]fludarabine PET/CT, five mice received rituximab (Mabthera, Roche) which was administrated by an *ip* injection (10 mg/kg, diluted in physiological saline, 500 μl). Five mice received injections of physiological saline alone (500 μl). Vehicle and rituximab treatments were performed thrice during the study (Additional file 1).

Tumoural growth was monitored by calliper measurements, three times weekly. The greatest longitudinal (d1) and transverse (d2) diameters were determined, and tumour volume was calculated by the modified ellipsoidal formula: tumour volume = ½ (d1 × d2^2^).

In order to perform a comparative study of the two tracers ([^18^F]fludarabine *vs*. [^18^F]FDG), additional eight mice were treated (three rituximab-treated and five vehicle-treated) at the same dates and under the same conditions as done in the sequential studies with [^18^F]fludarabine.

The behaviour of the mice during the experimental period was normal. Their body weights remained constant throughout the investigation (18.8 ± 0.8 g). Mice were maintained under isoflurane anaesthesia during radiotracer administration, scanning of either radiotracer (induction 5%, maintenance 2%, with 70% N_2_O/30% O_2_, Minerve anaesthesia system, Bioscan, France). Body temperature was maintained at 37°C using a feedback controlled system (Minerve, Bioscan, France).

Immediately after imaging studies were completed, the mice were sacrificed (cervical dislocation under deep anaesthesia). The complete tumour was carefully separated from muscle and skin and then excised.

### Imaging sessions

Firstly, [^18^F]fludarabine time-activity curves (TACs) were obtained over a 60-min period for tumour and skeletal muscle (non-target tissue) (*n* = 3) (Additional file 2).

For the sequential studies with [^18^F]fludarabine, the rituximab-treated (*n* = 5) and vehicle-treated (*n* = 5) mice were imaged thrice: a baseline scan (1 day before dosing) and two post-treatment scans (Additional file 1).

Additional mice (*n* = 8) were imaged with [^18^F]FDG only once to correspond to the post-treatment period of the last [^18^F]fludarabine scans. A comparative study was performed where the histological concordance was undertaken for each of the two radiotracers.

### PET/CT imaging

Images were acquired on an Inveon microPET/CT scanner (Siemens, Knoxville, USA) [8].

A CT scan was used for attenuation correction and localization of the tumour. The duration of the transmission scan was approximately 5 min. Acquisition parameters were: 80-kVp beam energy, 500-mAs current. The data were reconstructed into a 352 × 352 × 606 matrix images, corresponding to voxels of 0.022 × 0.022 × 0.022 mm^3^.

[^18^F]Fludarabine (11.21 ± 2.70 MBq in 100-μl physiological saline) was injected *via* the caudal vein, and PET images were acquired 40 to 60 min after radiotracer administration. [^18^F]FDG was injected with a similar activity (12.59 ± 1.20 MBq), and the PET scan was performed 70 to 90 min after radiotracer administration. The residual radioactivity in the syringe was measured to determine the received dose. An emission scan was acquired with default settings of coincidence timing window of 3.4 ns and an energy window of 350 to 650 keV. PET images were reconstructed with 3-dimensional maximum *a posteriori* (OSEM3D/MAP) reconstruction algorithm with 18 iterations (preceded by two OSEM3D iterations) with the beta parameter set to 0.2. Setting values of these parameters are discussed elsewhere [9]. The data were reconstructed into 128 × 128 × 159 matrix images, corresponding to a voxel of 0.776 × 0.776 × 0.796 mm^3^. Data were normalized and dead-time, random, scatter as well as attenuation corrections were applied.

### Analyses of image data

PMOD version 3.1 (PMOD Technologies, Ltd., Zurich, Switzerland) was used for image analysing and PET/CT co-registration.

Images were displayed as coronal, sagittal and transaxial slices, and the region of interest (ROI) was drawn manually on the coronal plane. The volume of interest (VOI), consisting of 2D ROIs and delineating the entire extent of the tumour, was defined on contiguous CT images and transposed to the PET images for quantification. Two methods of quantification were employed: total tumour-based (VOI_TOT_, encompassing the entire tumour) and a MAVT-based (VOI_MAVT_, delineating the metabolically active volume of the tumour). To define the MAVT, a threshold was applied to the drawn VOI_TOT_ in order to eliminate the pixels in which the values of intensity were below the activity in muscle (taken as the non-specific reference tissue). The segmentation was directed to find multiple disconnected contours if required. The VOI_MAVT_ was used for the data analyses of the sequential studies with [^18^F]fludarabine PET/CT, thus correlating the MAVT to the radiotracer accumulation (%ID). The VOI_TOT_ was used to correlate radiotracer uptake with histological quantification of whole sections in order to take into account the tumoural heterogeneity (non-lymphoid tissues in the tumour). Therefore, to compare the specificity of [^18^F]fludarabine to [^18^F]FDG in rituximab- and vehicle-treated animals, tracer concentration (%ID/ml) in the VOI_TOT_ was correlated to the percentage of viable lymphoid cells (in representative sections) as determined by the automatic quantification of the histological data, see below.

### Histopathological analyses

Paraffin-embedded tissue samples (fixed in 4% formation) were routinely processed. Paraffin blocks were used for haematoxylin-eosin-saffron (H.E.S.) staining. Conventional 5-μm-thick histological sections were obtained. Paraffin-embedded sections were deparaffinized and stained with CD20 (clone L26, 1:400 dilution), CD10 (clone 56C6, 1:30 dilution) and Bcl2 (clone 124, 1:100 dilution) in an automated immunohistochemistry processor (BenchMark XT; Ventana Medical Systems, Illkirch Graffenstaden, France) for antigen expression by validated staining protocols. The acquisition conditions of H.E.S. histological slides were standardized in order to generate colours of equal intensity and used in subsequent comparisons with the *in vivo* results.

The surface ratio between viable lymphoid tissue and whole histological section (total tissues) of each specimen was assessed by a computerized analysis (ImageJ, version 1.43, http://imagej.nih.gov/ij/). In brief, colour RGB images were deconvolved (red, green and blue), and the resulting monochrome images were thresholded. The R and G elements were used for segmentation of viable lymphoid and total tissues, respectively. This quantification was then correlated with PET/CT data (radiotracer concentration in tumour) [10].

### Statistical analyses

A *p* value <0.05 was considered statistically significant. In all analyses, normality and equality of variance were tested.

Pearson’s correlation coefficient was determined to evaluate the relationship between radiotracer uptake and tumour volume or histologically derived data (Prism 4.03, GraphPad Software, USA).

Repeated measures (RM) two-way ANOVA followed by Bonferroni post-tests determined the statistical difference in tumour volume between treated and non-treated animals (Sigma Plot 11.2, Jandel Scientific, Germany).

## Results

### Assessment of tumour volume by [^18^F]fludarabine PET/CT

**Figure 1:**
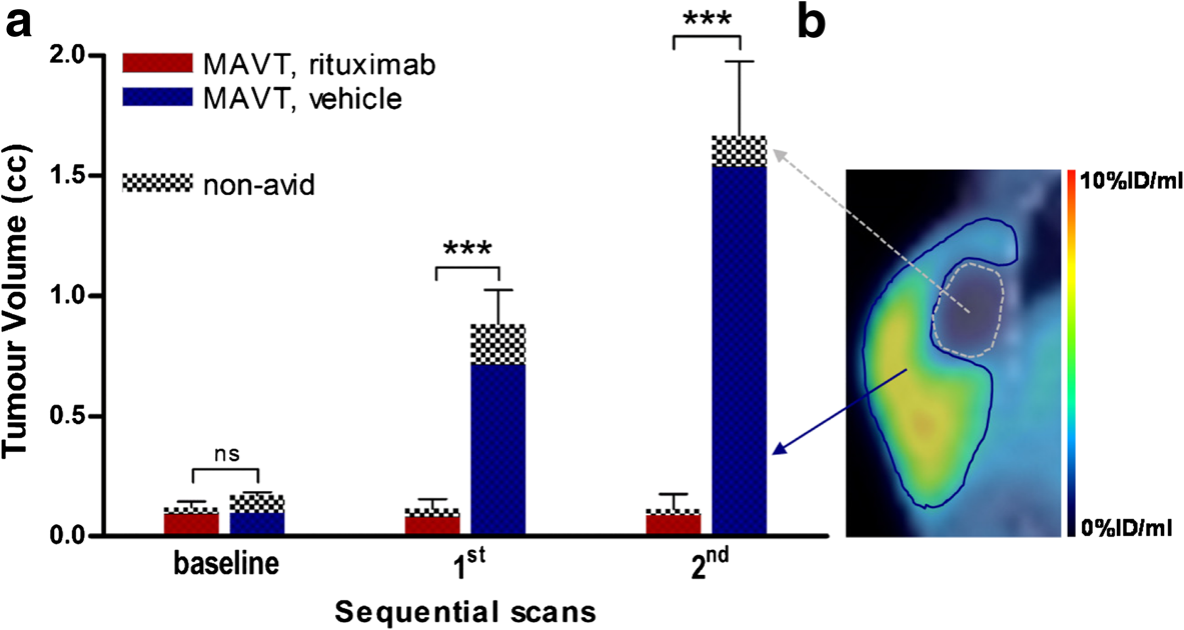
Volumetric assessment with [^18^F]fludarabine PET/CT and illustration of a delineation of MAVT. **(a)** Tumour growth in rituximab-treated (*n* = 5) and vehicle-treated mice (*n* = 5) - RM two-way ANOVA, *** *p* < 0.001. Error bars: ± standard error of the mean (SEM). **(b)** Contouring of metabolically active part of tumour (MAVT) based on segmentation with background (muscle) as threshold. The volume of non-avid tissues was assessed by subtracting the MAVT from the total tumour volume.

Total tumoural volume measurements were based on CT scans. At baseline, the volume was essentially the same in rituximab- and vehicle-treated mice (Figure 1a). Treatment with rituximab suppressed tumour growth in all treated mice, while in vehicle-treated mice the tumour volume continued to increase throughout the study (*p* < 0.001).

MAVT measurements, which were based on PET/CT scan, showed a similar evolution (Figure 1a). As mentioned previously, for the delineation of MAVT, a threshold was applied to the VOI_TOT_, drawn on the CT image and placed on the corresponding PET image, in order to eliminate those pixels in which the values of intensity were below the background level (Figure 1b).

### Sequential studies with [^18^F]fludarabine PET/CT

**Figure 2:**
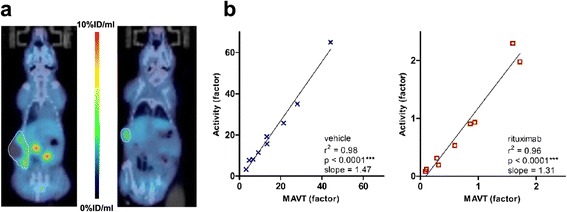
Illustrations of typical PET/CT scans and quantitative [^18^F]fludarabine PET/CT analysis. **(a)** Co-registered and fused coronal images for a vehicle-treated (to the left of the colour scale) and a rituximab-treated animal (to the right of the colour scale); the totality of the tumour is delineated on the illustrations. **(b)** Relationship between [^18^F]fludarabine accumulation [%ID] and tumour volume (vehicle-treated, *n* = 9: left graph and rituximab-treated, *n* = 9: right graph); PET quantification with VOI_MAVT_ based strictly on the viable lymphoid tissue. Data are displayed as a ratio of baseline (defined as 1). Correlation coefficients and significance were determined using Pearson’s test.

It was mentioned that the tumour volume continued to increase in the vehicle-treated mice throughout the study period (MAVT range, 0.1 to 1.54 cc). There was a significant correlation between the MAVT and the radiotracer accumulation (%ID) in those viable areas (*r*^2^ = 0.98, *p* < 0.0001) (Figure 2b); a similar relationship was observed in the rituximab-treated group, with Pearson’s test revealing a significant correlation between related data (*r*^2^ = 0.96, *p* < 0.0001).

### Comparative analyses of [^18^F]fludarabine and [^18^F]FDG

**Figure 3:**
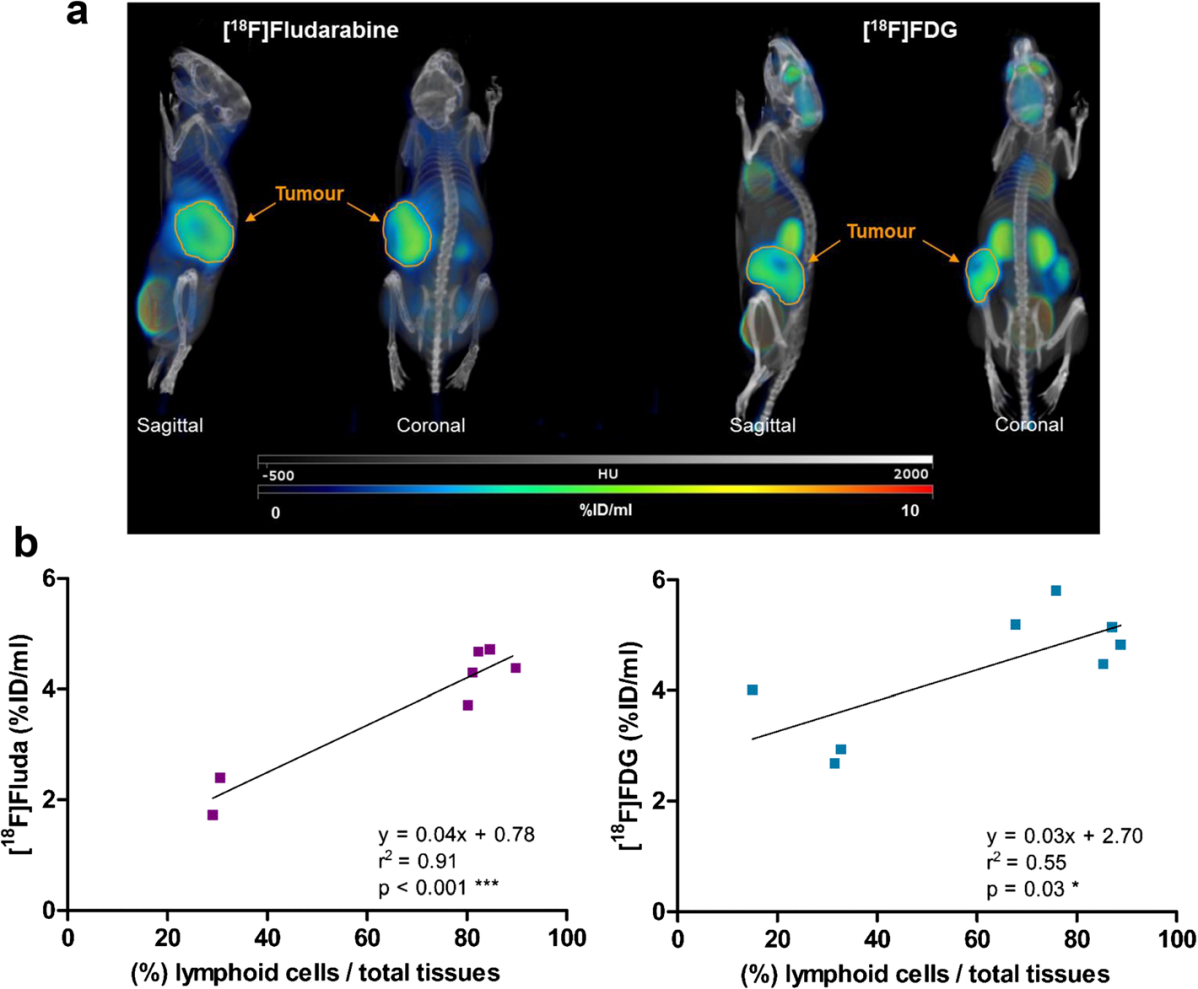
Illustration of coronal fused PET/CT images with [^18^F]fludarabine and [^18^F]FDG and quantitative comparative analysis. **(a)** Maximum intensity projections (3D rendering) highlighting the difference between these two radiotracers in terms of normal physiological uptake (e.g. brain, heart, etc.); the totality of the tumour is delineated on the illustrations. **(b)** Relationship between quantitative values extracted from PET/CT and histology; PET quantification with VOI_TOT_ taking into account the tumour heterogeneity. Total uptake [%ID/ml] of the tracer was correlated to the percentage of lymphoid cells in representative sections from each lymphoma as determined by automatic quantification of the histological slices ([^18^F]fludarabine, *n* = 7: left graph and [^18^F]FDG, *n* = 8: right graph). Correlation coefficients and significance were determined using Pearson’s test.

[^18^F]Fludarabine showed a great clearance from physiological tissues which resulted in a marked contrast between tumoural and normal tissues (Figure 3a).

A correlation analysis was performed between the histologically derived surface ratio of viable lymphoid cells to the total tissue mass and PET/CT-derived radiotracer concentrations (%ID/ml), in order to evaluate the pertinence of [^18^F]fludarabine and [^18^F]FDG as robust tracers. To obtain these correlations, the VOI_TOT_ was used for the PET scan analysis in order to take into account tumour heterogeneity. Correlation analyses revealed a powerful relationship between quantitative values extracted from [^18^F]fludarabine PET and histology (*r*^2^ = 0.91, *p* < 0.001); the comparable results for [^18^F]FDG were at the limit of statistical significance (*r*^2^ = 0.55, *p* = 0.03) (Figure 3b).

### Histopathology of tumours

**Figure 4:**
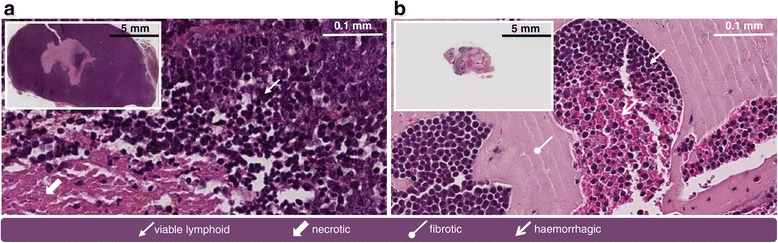
Histological illustrations. Histological section (H.E.S.) and microscopic view (SlidePath Gateway 2.0, Leica Biosystems, Germany) of a tumour from **(a)** vehicle-treated animal and **(b)** rituximab-treated animal.

The immunohistochemical characteristics of the engrafted tumours, i.e. overexpression of the proteins CD20, CD10, and Bcl2, were similar in both rituximab- and vehicle-treated mice. These features are the hallmarks of the human lymphoma cell line. These similarities strongly support the origin of the large B-cell lymphoma from a follicular lymphoma in that CD10 and Bcl2 displayed a diffuse positive reaction. In vehicle-treated mice, the tumoural tissue was principally composed of lymphoma cells and contiguous necrotic areas (Figure 4); in rituximab-treated mice, in addition to lymphoid clusters and necrotic cells, the analysis revealed fibrotic and haemorrhagic areas.

## Discussion

The goal of this study was to investigate the eventual usefulness of surveillance [^18^F]fludarabine PET/CT, based on a xenograft model of a follicular lymphoma. We assessed the robustness of [^18^F]fludarabine during rituximab therapy and performed an inter-tracer comparison between [^18^F]fludarabine and [^18^F]FDG. This investigation is the logical follow-up to our earlier publication in which many other preclinical features of [^18^F]fludarabine were described. The choice of the follicular lymphoma model was based on, primarily, its efficacious response to treatment with the anti-CD20 antibody which alone, or in combination with other chemotherapeutic agents, is useful in the treatment of low-grade NHL [11]*.* As follicular lymphoma is almost universally FDG avid, we initiated comparative analysis between [^18^F]fludarabine and [^18^F]FDG with respect to their concordance with histologically derived data. Although [^18^F]FDG-PET has proven helpful in the diagnosis and therapy follow-up in patients with lymphoma, the specificity of [^18^F]FDG uptake has been brought into question because of its behaviour similar to glucose metabolism, which may indiscriminately increase in benign processes such as inflammation or infection [3-5].

In a previous study in SCID mice bearing a human follicular lymphoma (RL-7), we demonstrated a marked difference in the behaviour of [^18^F]fludarabine in comparison with [^18^F]FDG immediately after their administration, a difference that favoured [^18^F]fludarabine. The accumulation of the latter tracer in the tumour increased rapidly over the first 20 min and subsequently plateaued in contrast to that of [^18^F]FDG for which no plateau could be determined, even at the final phase of the examination. The tissue uptake of [^18^F]FDG is highly variable, with concentrations not reaching a plateau for up to 4 to 6 h in some tumours [12]. As previously described, [^18^F]fludarabine shows a great washout from normal tissues which, in turn, results in a pronounced contrast between tumoural and normal tissues.

In positron emission tomography, the delineation method of the target volume on PET images is crucial for quantification. A fundamental biological question, underlying choices of regions of interest in PET analysis, is whether either the total tumour volume or the maximally metabolically active portion of the tumour is the most important [13,14]. In this present investigation, we have firstly measured the uptake in VOI_TOT_ which was then compared to histological data. The VOI_MAVT_ was calculated for the surveillance of the lymphoma considering only its metabolically active portion. A wide variety of methods exist to delineate the MAVT [12-16] such as isocontours based on a fixed percentage of the maximal pixel in the tumour or adaptive isocontours based on a threshold set to background. This latter is an attractive concept if the uptake in the background (normal organ) is stable over time [13]. In our study, ‘all above background’ was considered MAVT, with skeletal muscle activity set as the threshold, which - as revealed by RM two-way ANOVA - was stable over sequential PET scans with no significant main effects of group (*F*_1.29_ = 0.14), of time (*F*_2.29_ = 0.73) and with no significant interaction (*F*_2.29_ = 3.38, *p* = ns for all).

Based on either calliper or volume by imaging measurements, tumour growth was abolished (despite inter-animal variability) in all rituximab-treated animals, while in vehicle-treated mice the time to double the tumour volume was 6 days, as analysed by their exponential growth rate.

In the vehicle-treated mice, where the tumour volume (and also the MAVT) continued to increase throughout the experiment, there was a significant relationship between the volume of the metabolically active part of the tumour and the [^18^F]fludarabine accumulation in those viable tissues. This relationship was equally significant in the rituximab-treated mice when correlating the MAVT of the residual tissue with the tracer accumulation. The presence of lymphoid tissue at the end of imaging sessions was confirmed histologically, indicating therefore the ability of [^18^F]fludarabine PET/CT to detect persistent viable tumour areas.

It is known that false positives occur with clinical FDG-PET in diffuse large B-cell lymphoma with rituximab therapy [3]. Indeed, rituximab administration may induce necrosis and relatively long-lasting inflammatory changes in lymphoid tissues, resulting in a non-specific consumption of [^18^F]FDG [17-20]. Although FDG-PET has proven to be sensitive for surveillance of lymphoma, its specificity appears to be insufficient, resulting in a disturbingly large number of false-positive results.

To address the issue of radiotracer specificity, we determined the relationship between PET/CT and histologically derived data by correlating the tracer uptake in the totality of the tumour to the percentage of lymphoid cells. This approach revealed a weaker correlation for [^18^F]FDG when compared to [^18^F]fludarabine, presumably due to a nonspecific accumulation of [^18^F]FDG in glucose-consuming inflammatory or stromal cells [4,21-23].

In addition, as the cellular uptake of fludarabine is cell-cycle independent [24], [^18^F]fludarabine PET/CT is a method that holds promise for imaging lymphoid neoplasms characterized by low mitotic activity that include chronic lymphocytic leukaemia. This latter typically has weak [^18^F]FDG avidity, and the addition of the [^18^F]FDG PET component has not been shown to improve the usefulness of CT scans alone in the management of this specific cohort of patients.

## Conclusions

In conclusion, the results of present study, coherent with our previous investigations, demonstrate several major characteristics that speak in favour of this novel radiopharmaceutical agent. Firstly, the use of [^18^F]fludarabine signals a marked tumour/normal tissue contrast and also demonstrates considerable specificity. Furthermore, the rituximab treatment did not interfere with [^18^F]fludarabine uptake in the present model of lymphoma. Accordingly, [^18^F]fludarabine may be considered a promising approach for detecting the persistence of follicular lymphoid tissues during or after rituximab-like treatment. Further investigations are required to extend this conclusion to other specific types of lymphoma or treatment. Indeed, the accurate surveillance by PET can help or change the treatment recommendations, thus avoiding unnecessarily biopsies consequent to false-positive imaging studies [25]. Of note, the use of [^18^F]fludarabine in fludarabine-treated subjects remains to be tested. Since, however, fludarabine is no longer detectable 24 h after the last dose [26], [^18^F]fludarabine uptake, when assessed after that period of time, should not be affected. These encouraging findings suggest that [^18^F]fludarabine PET/CT might well be an innovative approach for surveillance of lymphoma. This positron-emitting biomarker could be also used for tumour chemosensitivity assays in order to identify fludarabine-avid tumours.

## Additional files

Additional file 1:
**Tumour volume growth curves (calliper measurements) of CB17-SCID mice bearing subcutaneous DOHH-2 human B cell lymphoma.** Mice were SC injected with tumour cells at day 0. Mice were treated with vehicle or rituximab (10 mg/kg, *ip*) at day 22, 29 and 36. Red arrows represent treatment days. t_0_ (day 21), t_1_ (day 34) and t_2_ (day 42) represent [^18^F]fludarabine PET/CT imaging days for treated animals at baseline (1 day before dosing) and follow-up scans, respectively. t_0_′ (day 23), t_1_′ (day 37) and t_2_′ (day 44) represent [^18^F]fludarabine PET/CT imaging days for vehicle animals at baseline and follow-up scans, respectively. Error bars: ± SEM. Additional file 2:
**Time activity curves (TAC) of the tumour and muscle (non-target tissue) with [18F]fludarabine.** TACs were determined on dynamic PET images (*n* = 3). On PET/CT fused data, blue and yellow arrows indicate tumour and muscle, respectively. Error bars: ± SEM.
