# Supplementary material for: Evaluation of the specificity of [18F]fludarabine PET/CT in a xenograft model of follicular lymphoma: comparison with [18F]FDG and impact of rituximab therapy
Source: EJNMMI Res. 2015 Apr 14;5:23. doi: 10.1186/s13550-015-0101-7 (PMC4414862; doi:10.1186/s13550-015-0101-7)

Additional file 1 Tumour volume growth curves (calliper measurements) of CB17 SCID mice bearing subcutaneous DOHH2 human B cell lymphoma. Mice were SC injected with tumour cells at day 0. Mice were treated with vehicle or rituximab (10 mg/kg, *ip*) at day 22, 29 and 36. Red arrows represent treatment days. t_0 (day 21), t_1 (day 34) and t_2 (day 42) represent [^{18}F]fludarabine PET/CT imaging days for treated animals at baseline (1 day before dosing) and follow-up scans, respectively. t_0' (day 23), t_1' (day 37) and t_2' (day 44) represent [^{18}F]fludarabine PET/CT imaging days for vehicle animals at baseline and follow-up scans, respectively. Error bars: ± SEM.

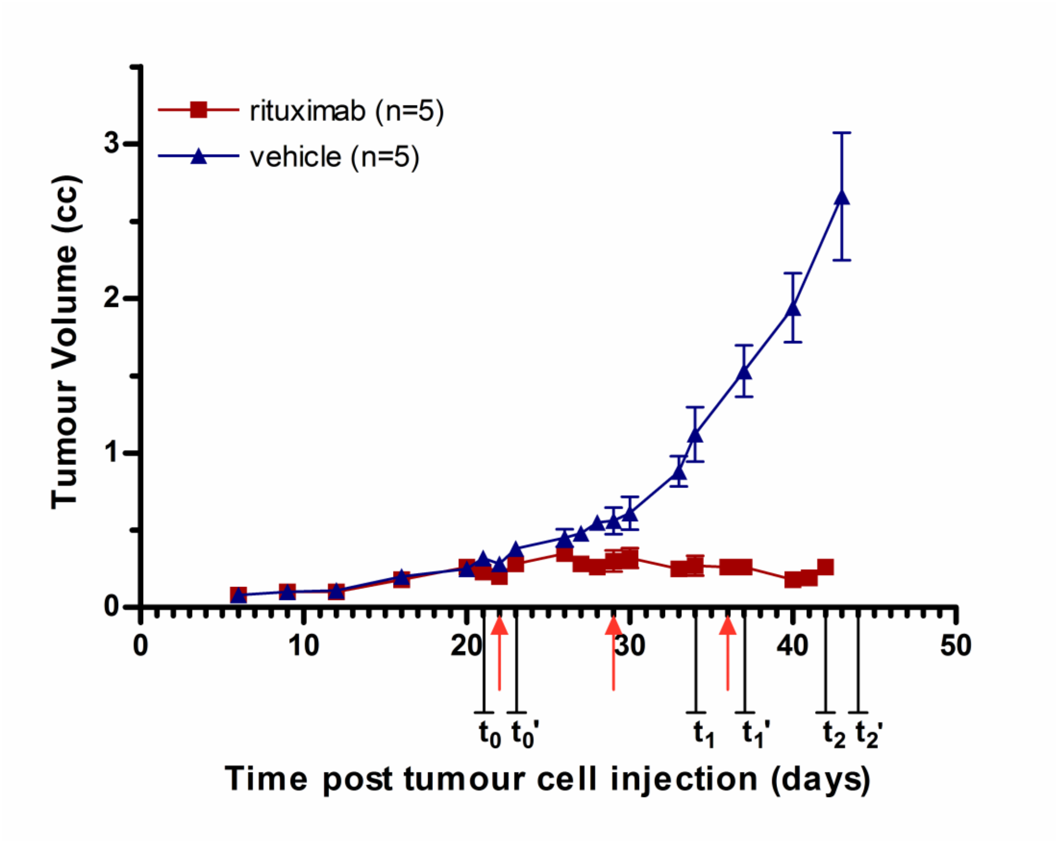

Supplement: Additional file 1: — Tumour volume growth curves (calliper measurements) of CB17-SCID mice bearing subcutaneous DOHH-2 human B cell lymphoma. Mice were SC injected with tumour cells at day 0. Mice were treated with vehicle or rituximab (10 mg/kg, ip) at day 22, 29 and 36. Red arrows represent treatment days. t0 (day 21), t1 (day 34) and t2 (day 42) represent [18F]fludarabine PET/CT imaging days for treated animals at baseline (1 day before dosing) and follow-up scans, respectively. t0′ (day 23), t1′ (day 37) and t2′ (day 44) represent [18F]fludarabine PET/CT imaging days for vehicle animals at baseline and follow-up scans, respectively. Error bars: ± SEM. [file 13550_2015_101_MOESM1_ESM.pdf]
