# Supplementary material for: Evaluation of the specificity of [18F]fludarabine PET/CT in a xenograft model of follicular lymphoma: comparison with [18F]FDG and impact of rituximab therapy
Source: EJNMMI Res. 2015 Apr 14;5:23. doi: 10.1186/s13550-015-0101-7 (PMC4414862; doi:10.1186/s13550-015-0101-7)

Additional file 2 Time activity curves (TAC) of the tumour and muscle (non-target tissue) with [^{18}F]fludarabine. TACs were determined on dynamic PET images (n = 3). On PET/CT fused data, blue and yellow arrows indicate tumor and muscle, respectively. Error bars: ± SEM.

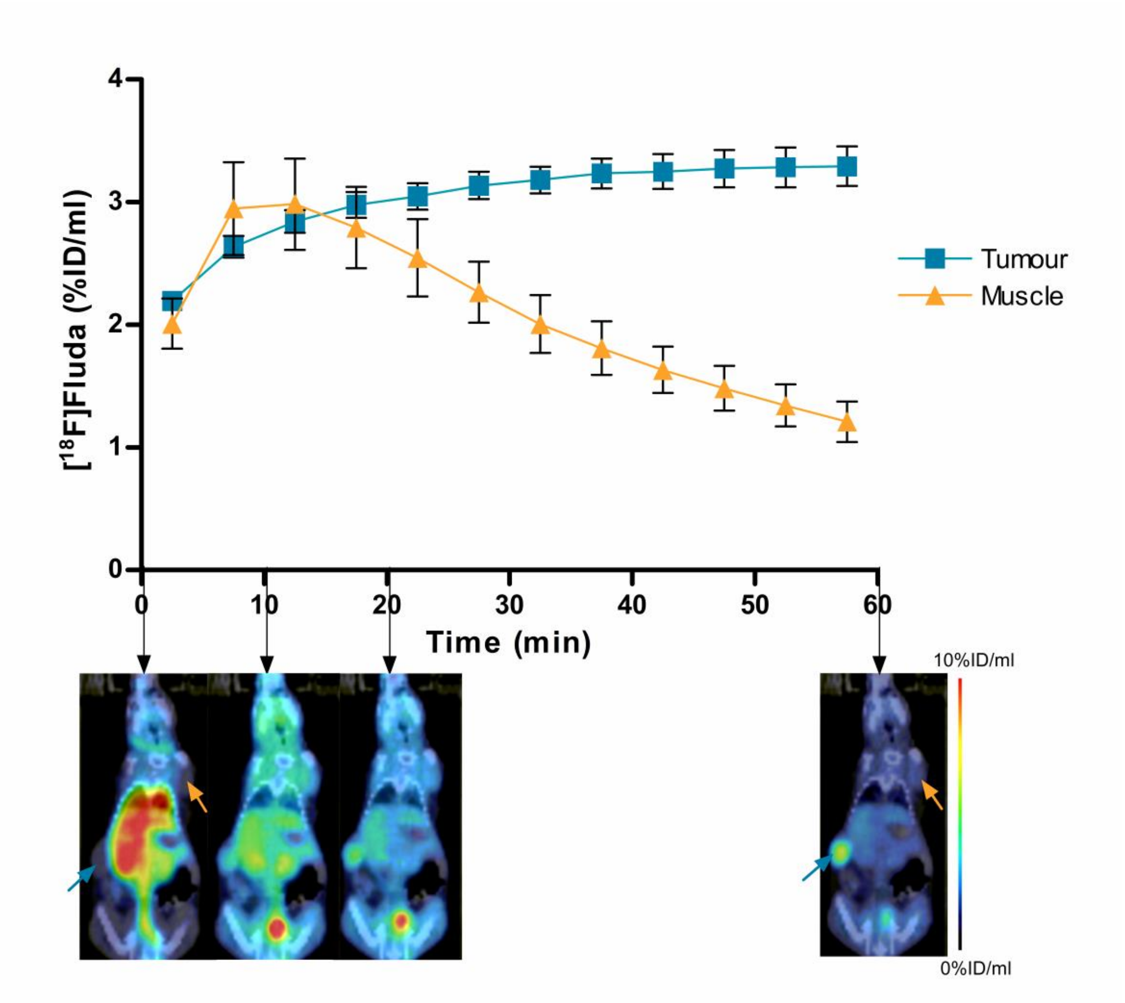

Supplement: Additional file 2: — Time activity curves (TAC) of the tumour and muscle (non-target tissue) with [18F]fludarabine. TACs were determined on dynamic PET images (n = 3). On PET/CT fused data, blue and yellow arrows indicate tumour and muscle, respectively. Error bars: ± SEM. [file 13550_2015_101_MOESM2_ESM.pdf]
